# Diethyl 2-{[3-(2-meth­oxy­benz­yl)thio­phen-2-yl]methyl­idene}malonate

**DOI:** 10.1107/S1600536811022525

**Published:** 2011-06-18

**Authors:** S. Ranjith, K. Sakthi Murugesan, A. Subbiah Pandi, V. Dhayalan, A. K. Mohana Krishnan

**Affiliations:** a Department of Physics, Presidency College (Autonomous), Chennai 600 005, India; b Department of Organic Chemistry, University of Madras, Guindy Campus, Chennai 600 025, India

## Abstract

In the title compound, C_20_H_22_O_5_S, the dihedral angle between the mean planes through the thio­phene and benzene rings is 75.2 (1)°. The meth­oxy group is essentially coplanar with the benzene ring, the largest deviation from the mean plane being 0.019 (2) Å for the O atom. The malonate group assumes an extended conformation.

## Related literature

For the biological activities of thio­phene derivatives, see: Bonini *et al.* (2005 ▶); Brault *et al.* (2005 ▶); Isloora *et al.* (2010 ▶); Xia *et al.* (2010 ▶). For a similar thio­phene structure, see: Dufresne & Skene (2010 ▶).
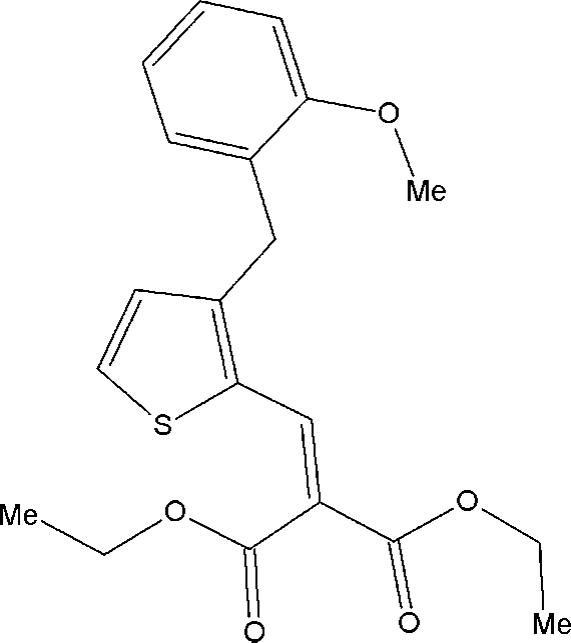


## Experimental

### 

#### Crystal data

C_20_H_22_O_5_S
*M* *_r_* = 374.44
Orthorhombic, 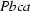

*a* = 8.1680 (2) Å
*b* = 16.4046 (4) Å
*c* = 28.8651 (7) Å
*V* = 3867.72 (16) Å^3^
*Z* = 8
Mo *K*α radiation
μ = 0.19 mm^−1^
*T* = 293 K
0.25 × 0.22 × 0.19 mm

#### Data collection

Bruker APEXII CCD area detector diffractometer
Absorption correction: multi-scan (*SADABS*; Sheldrick, 1996 ▶) *T*_min_ = 0.953, *T*_max_ = 0.964
35690 measured reflections
3695 independent reflections
2687 reflections with *I* > 2σ(*I*)
*R*_int_ = 0.053

#### Refinement

*R*[*F* ^2^ > 2σ(*F* ^2^)] = 0.042
*wR*(*F* ^2^) = 0.145
*S* = 1.02
3695 reflections
238 parameters
H-atom parameters constrained
Δρ_max_ = 0.30 e Å^−3^
Δρ_min_ = −0.21 e Å^−3^

### 

Data collection: *APEX2* (Bruker, 2007 ▶); cell refinement: *APEX2*; data reduction: *SAINT* (Bruker, 2007 ▶); program(s) used to solve structure: *SHELXS97* (Sheldrick, 2008 ▶); program(s) used to refine structure: *SHELXL97* (Sheldrick, 2008 ▶); molecular graphics: *ORTEP-3* (Farrugia, 1997 ▶); software used to prepare material for publication: *SHELXL97* and *PLATON* (Spek, 2009 ▶).

## Supplementary Material

Crystal structure: contains datablock(s) global, I. DOI: 10.1107/S1600536811022525/nk2101sup1.cif

Structure factors: contains datablock(s) I. DOI: 10.1107/S1600536811022525/nk2101Isup2.hkl

Supplementary material file. DOI: 10.1107/S1600536811022525/nk2101Isup3.cml

Additional supplementary materials:  crystallographic information; 3D view; checkCIF report

## Figures and Tables

**Table 1:** Hydrogen-bond geometry (Å, °)

*D*—H⋯*A*	*D*—H	H⋯*A*	*D*⋯*A*	*D*—H⋯*A*
C7—H7⋯O2^i^	0.93	2.55	3.429 (3)	159

Symmetry code: (i) 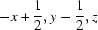.

**Fig. 1.**
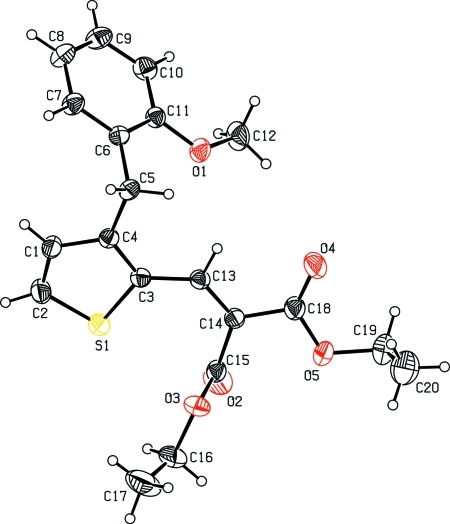
The structure of the title compound with displacement ellipsoids at the 30% probability level.

## supplementary crystallographic information

### Comment

Thiophene derivatives exhibit anti-HIVPR inhibition (Bonini *et al.*, 2005) and antibreast cancer (Brault *et al.*, 2005) activity. In addition, some of the benzo[*b*]thiophene derivatives show significant antimicrobial and anti-inflammatory activity (Isloora *et al.*, 2010). Thiophene derivates have been viewed as significant compounds for applications in many fields (Xia *et al.*, 2010). Against this background, and in order to obtain detailed information on molecular conformations in the solid state, an X-ray study of the title compound was carried out.

X-Ray analysis confirms the molecular structure and atom connectivity as illustrated in Fig. 1. The bond lengths and angles in (Fig. 1) agree with those observed in other thiophene derivative (Dufresne & Skene, 2010). The thiophene ring system make the dihedral angle of 75.2 (1)° with respect to benzene ring. The atom O1 is deviated by 0.019 (2) Å from the least-squares plane of the benzene ring. The malonate group assumes an extended conformation as can be seen from torsion angles C14—C18—O5—C19 of 178.3 (2)° and C14—C15—O3—C16 of 173.9 (2)°.

### Experimental

To a solution of diethyl-2-((3-(bromomethyl)thiophen-2-yl)methylene)malonate (2.88 mmol) in dry dichloroethane (15 ml), anhydrous ZnBr_2_ (2.84 mmol) and anisole (3.17 mmol) were added. The reaction mixture was stirred at room temperature for 9 h and then refluxed for 1 h under N_2_ atmosphere. The solvent was removed and the residue was quenched with ice–water (50 ml) containing 1 ml of Conc. HCl, extracted with chloroform (3 × 10 ml) and dried (Na_2_SO_4_). Removal of solvent followed by flash column chromatographic purification (n-hexane/ethyl acetate 98:2) led to the isolation of diethyl-2-((3-(2-methoxybenzyl)thiophen-2-yl) methylene)malonate as a colorless crystal. Single crystals suitable for X-ray diffraction were obtained by slow evaporation of a solution of the title compound in methanol at room temperature.

### Refinement

All H atoms were fixed geometrically and allowed to ride on their parent C atoms, with C—H distances fixed in the range 0.93–0.97 Å with *U*_iso_(H) = 1.5*U*_eq_(C) for methyl H 1.2*U*_eq_(C) for other H atoms.

### Crystal data

**Table:**

C_20_H_22_O_5_S	*F*(000) = 1584
*M**_r_* = 374.44	*D*_x_ = 1.286 Mg m^−^^3^
Orthorhombic, *P**b**c**a*	Mo *K*α radiation, λ = 0.71073 Å
Hall symbol: -P 2ac 2ab	Cell parameters from 3696 reflections
*a* = 8.1680 (2) Å	θ = 1.4–25.8°
*b* = 16.4046 (4) Å	µ = 0.19 mm^−^^1^
*c* = 28.8651 (7) Å	*T* = 293 K
*V* = 3867.72 (16) Å^3^	Block, white
*Z* = 8	0.25 × 0.22 × 0.19 mm

### Data collection

**Table:**

Bruker APEXII CCD area detector diffractometer	3695 independent reflections
Radiation source: fine-focus sealed tube	2687 reflections with *I* > 2σ(*I*)
graphite	*R*_int_ = 0.053
ω and φ scans	θ_max_ = 25.8°, θ_min_ = 2.5°
Absorption correction: multi-scan (*SADABS*; Sheldrick, 1996)	*h* = −9→9
*T*_min_ = 0.953, *T*_max_ = 0.964	*k* = −20→19
35690 measured reflections	*l* = −35→35

### Refinement

**Table:**

Refinement on *F*^2^	Primary atom site location: structure-invariant direct methods
Least-squares matrix: full	Secondary atom site location: difference Fourier map
*R*[*F*^2^ > 2σ(*F*^2^)] = 0.042	Hydrogen site location: inferred from neighbouring sites
*wR*(*F*^2^) = 0.145	H-atom parameters constrained
*S* = 1.02	*w* = 1/[σ^2^(*F*_o_^2^) + (0.0896*P*)^2^ + 0.4403*P*] where *P* = (*F*_o_^2^ + 2*F*_c_^2^)/3
3695 reflections	(Δ/σ)_max_ = 0.001
238 parameters	Δρ_max_ = 0.30 e Å^−^^3^
0 restraints	Δρ_min_ = −0.21 e Å^−^^3^

### Special details

**Table:**

Geometry. All e.s.d.'s (except the e.s.d. in the dihedral angle between two l.s. planes) are estimated using the full covariance matrix. The cell e.s.d.'s are taken into account individually in the estimation of e.s.d.'s in distances, angles and torsion angles; correlations between e.s.d.'s in cell parameters are only used when they are defined by crystal symmetry. An approximate (isotropic) treatment of cell e.s.d.'s is used for estimating e.s.d.'s involving l.s. planes.
Refinement. Refinement of *F*^2^ against ALL reflections. The weighted *R*-factor *wR* and goodness of fit *S* are based on *F*^2^, conventional *R*-factors *R* are based on *F*, with *F* set to zero for negative *F*^2^. The threshold expression of *F*^2^ > σ(*F*^2^) is used only for calculating *R*-factors(gt) *etc*. and is not relevant to the choice of reflections for refinement. *R*-factors based on *F*^2^ are statistically about twice as large as those based on *F*, and *R*-factors based on ALL data will be even larger.

### Fractional atomic coordinates and isotropic or equivalent isotropic displacement parameters (Å^2^)

**Table:**

	*x*	*y*	*z*	*U*_iso_*/*U*_eq_	
C1	0.2480 (3)	0.84683 (13)	0.40960 (7)	0.0502 (5)	
H1	0.2853	0.7955	0.4182	0.060*	
C2	0.0917 (3)	0.87086 (14)	0.41448 (8)	0.0577 (6)	
H2	0.0097	0.8382	0.4270	0.069*	
C3	0.2653 (2)	0.97752 (12)	0.37999 (6)	0.0406 (5)	
C4	0.3501 (2)	0.90728 (11)	0.39004 (6)	0.0391 (4)	
C5	0.5308 (2)	0.89232 (12)	0.38274 (6)	0.0416 (5)	
H5A	0.5455	0.8420	0.3656	0.050*	
H5B	0.5764	0.9363	0.3644	0.050*	
C6	0.6220 (2)	0.88675 (12)	0.42814 (6)	0.0392 (4)	
C7	0.6554 (3)	0.81224 (12)	0.44834 (7)	0.0499 (5)	
H7	0.6246	0.7646	0.4332	0.060*	
C8	0.7338 (3)	0.80722 (15)	0.49063 (8)	0.0605 (6)	
H8	0.7551	0.7566	0.5039	0.073*	
C9	0.7800 (3)	0.87723 (15)	0.51296 (7)	0.0600 (6)	
H9	0.8333	0.8739	0.5414	0.072*	
C10	0.7487 (3)	0.95238 (14)	0.49386 (7)	0.0541 (6)	
H10	0.7799	0.9996	0.5094	0.065*	
C11	0.6703 (2)	0.95747 (11)	0.45135 (7)	0.0415 (5)	
C12	0.6731 (3)	1.10362 (14)	0.45138 (9)	0.0702 (7)	
H12A	0.6190	1.1058	0.4809	0.105*	
H12B	0.6364	1.1481	0.4325	0.105*	
H12C	0.7893	1.1076	0.4559	0.105*	
C13	0.3340 (2)	1.05129 (11)	0.36133 (6)	0.0410 (5)	
H13	0.4477	1.0519	0.3598	0.049*	
C14	0.2619 (2)	1.11897 (12)	0.34583 (6)	0.0418 (5)	
C15	0.0810 (2)	1.13214 (12)	0.34553 (7)	0.0467 (5)	
C16	−0.1688 (3)	1.09125 (18)	0.31161 (12)	0.0825 (9)	
H16A	−0.2077	1.1441	0.3013	0.099*	
H16B	−0.2117	1.0811	0.3424	0.099*	
C17	−0.2233 (4)	1.0273 (3)	0.27952 (11)	0.1122 (13)	
H17A	−0.1722	1.0351	0.2499	0.168*	
H17B	−0.3401	1.0300	0.2761	0.168*	
H17C	−0.1933	0.9748	0.2916	0.168*	
C18	0.3658 (3)	1.18679 (13)	0.32912 (8)	0.0541 (6)	
C19	0.3623 (4)	1.31693 (16)	0.29168 (9)	0.0758 (8)	
H19A	0.2947	1.3649	0.2963	0.091*	
H19B	0.4635	1.3244	0.3088	0.091*	
C20	0.3981 (4)	1.3071 (2)	0.24258 (10)	0.0962 (10)	
H20A	0.4699	1.2614	0.2383	0.144*	
H20B	0.4500	1.3556	0.2311	0.144*	
H20C	0.2980	1.2980	0.2259	0.144*	
O1	0.63527 (19)	1.02826 (8)	0.42901 (5)	0.0542 (4)	
O2	0.0111 (2)	1.17398 (10)	0.37271 (7)	0.0752 (5)	
O3	0.00937 (18)	1.08978 (10)	0.31226 (5)	0.0587 (4)	
O4	0.5109 (2)	1.18958 (11)	0.33296 (8)	0.0910 (7)	
O5	0.2766 (2)	1.24465 (9)	0.30883 (5)	0.0639 (5)	
S1	0.06061 (7)	0.96745 (4)	0.39524 (2)	0.0526 (2)	

### Atomic displacement parameters (Å^2^)

**Table:**

	*U*^11^	*U*^22^	*U*^33^	*U*^12^	*U*^13^	*U*^23^
C1	0.0581 (14)	0.0429 (11)	0.0496 (11)	−0.0054 (10)	−0.0043 (10)	0.0073 (9)
C2	0.0579 (15)	0.0586 (14)	0.0565 (13)	−0.0150 (12)	0.0021 (11)	0.0091 (11)
C3	0.0398 (11)	0.0421 (11)	0.0399 (10)	0.0006 (8)	−0.0028 (8)	−0.0007 (8)
C4	0.0450 (12)	0.0387 (10)	0.0338 (9)	−0.0009 (8)	−0.0038 (8)	−0.0015 (8)
C5	0.0489 (12)	0.0380 (10)	0.0378 (10)	0.0053 (9)	−0.0007 (9)	−0.0010 (8)
C6	0.0382 (10)	0.0396 (10)	0.0397 (10)	0.0030 (8)	0.0012 (8)	−0.0015 (8)
C7	0.0595 (14)	0.0385 (11)	0.0516 (12)	0.0054 (10)	−0.0067 (10)	−0.0025 (9)
C8	0.0754 (16)	0.0534 (14)	0.0526 (13)	0.0114 (12)	−0.0090 (11)	0.0089 (10)
C9	0.0710 (16)	0.0669 (16)	0.0422 (12)	0.0082 (13)	−0.0118 (11)	0.0015 (11)
C10	0.0600 (14)	0.0544 (14)	0.0478 (12)	−0.0047 (10)	−0.0056 (11)	−0.0106 (10)
C11	0.0420 (11)	0.0369 (10)	0.0457 (11)	0.0004 (8)	0.0015 (9)	−0.0011 (8)
C12	0.0827 (18)	0.0391 (12)	0.0889 (18)	−0.0098 (12)	−0.0012 (14)	−0.0097 (12)
C13	0.0364 (10)	0.0416 (11)	0.0450 (11)	0.0006 (8)	0.0001 (8)	−0.0002 (8)
C14	0.0389 (11)	0.0385 (10)	0.0480 (11)	0.0017 (8)	0.0007 (9)	−0.0007 (8)
C15	0.0447 (12)	0.0361 (10)	0.0592 (13)	0.0028 (9)	0.0043 (10)	0.0044 (10)
C16	0.0380 (13)	0.088 (2)	0.121 (2)	0.0024 (13)	−0.0115 (14)	0.0139 (18)
C17	0.070 (2)	0.178 (4)	0.088 (2)	−0.035 (2)	−0.0234 (17)	0.002 (2)
C18	0.0474 (14)	0.0444 (12)	0.0706 (14)	0.0025 (10)	0.0056 (11)	0.0067 (11)
C19	0.096 (2)	0.0568 (15)	0.0747 (17)	−0.0089 (14)	0.0089 (15)	0.0113 (13)
C20	0.111 (3)	0.101 (2)	0.0764 (19)	−0.0050 (19)	0.0193 (17)	0.0097 (17)
O1	0.0689 (10)	0.0344 (7)	0.0593 (9)	−0.0046 (7)	−0.0092 (7)	−0.0008 (6)
O2	0.0597 (10)	0.0637 (11)	0.1023 (13)	0.0107 (9)	0.0193 (10)	−0.0254 (10)
O3	0.0378 (8)	0.0702 (10)	0.0681 (10)	0.0033 (7)	−0.0059 (7)	−0.0051 (8)
O4	0.0428 (10)	0.0703 (12)	0.160 (2)	−0.0049 (9)	0.0016 (11)	0.0353 (12)
O5	0.0642 (10)	0.0492 (9)	0.0782 (11)	−0.0018 (8)	−0.0022 (8)	0.0227 (8)
S1	0.0422 (3)	0.0553 (4)	0.0603 (4)	−0.0012 (2)	0.0033 (2)	0.0067 (3)

### Geometric parameters (Å, °)

**Table:**

C1—C2	1.344 (3)	C12—H12B	0.9600
C1—C4	1.413 (3)	C12—H12C	0.9600
C1—H1	0.9300	C13—C14	1.334 (3)
C2—S1	1.698 (2)	C13—H13	0.9300
C2—H2	0.9300	C14—C18	1.480 (3)
C3—C4	1.375 (3)	C14—C15	1.493 (3)
C3—C13	1.439 (3)	C15—O2	1.189 (2)
C3—S1	1.737 (2)	C15—O3	1.322 (3)
C4—C5	1.511 (3)	C16—O3	1.456 (3)
C5—C6	1.510 (3)	C16—C17	1.469 (4)
C5—H5A	0.9700	C16—H16A	0.9700
C5—H5B	0.9700	C16—H16B	0.9700
C6—C7	1.381 (3)	C17—H17A	0.9600
C6—C11	1.396 (3)	C17—H17B	0.9600
C7—C8	1.381 (3)	C17—H17C	0.9600
C7—H7	0.9300	C18—O4	1.192 (3)
C8—C9	1.370 (3)	C18—O5	1.332 (3)
C8—H8	0.9300	C19—C20	1.456 (4)
C9—C10	1.375 (3)	C19—O5	1.463 (3)
C9—H9	0.9300	C19—H19A	0.9700
C10—C11	1.387 (3)	C19—H19B	0.9700
C10—H10	0.9300	C20—H20A	0.9600
C11—O1	1.359 (2)	C20—H20B	0.9600
C12—O1	1.429 (3)	C20—H20C	0.9600
C12—H12A	0.9600		
			
C2—C1—C4	113.4 (2)	H12B—C12—H12C	109.5
C2—C1—H1	123.3	C14—C13—C3	130.83 (19)
C4—C1—H1	123.3	C14—C13—H13	114.6
C1—C2—S1	112.43 (17)	C3—C13—H13	114.6
C1—C2—H2	123.8	C13—C14—C18	118.84 (18)
S1—C2—H2	123.8	C13—C14—C15	123.97 (18)
C4—C3—C13	125.96 (18)	C18—C14—C15	117.16 (17)
C4—C3—S1	110.60 (15)	O2—C15—O3	124.75 (19)
C13—C3—S1	123.39 (15)	O2—C15—C14	123.7 (2)
C3—C4—C1	112.01 (18)	O3—C15—C14	111.48 (17)
C3—C4—C5	126.77 (17)	O3—C16—C17	107.4 (2)
C1—C4—C5	121.21 (18)	O3—C16—H16A	110.2
C6—C5—C4	111.75 (15)	C17—C16—H16A	110.2
C6—C5—H5A	109.3	O3—C16—H16B	110.2
C4—C5—H5A	109.3	C17—C16—H16B	110.2
C6—C5—H5B	109.3	H16A—C16—H16B	108.5
C4—C5—H5B	109.3	C16—C17—H17A	109.5
H5A—C5—H5B	107.9	C16—C17—H17B	109.5
C7—C6—C11	118.48 (17)	H17A—C17—H17B	109.5
C7—C6—C5	121.15 (17)	C16—C17—H17C	109.5
C11—C6—C5	120.34 (17)	H17A—C17—H17C	109.5
C6—C7—C8	121.2 (2)	H17B—C17—H17C	109.5
C6—C7—H7	119.4	O4—C18—O5	123.9 (2)
C8—C7—H7	119.4	O4—C18—C14	124.7 (2)
C9—C8—C7	119.6 (2)	O5—C18—C14	111.45 (18)
C9—C8—H8	120.2	C20—C19—O5	109.6 (2)
C7—C8—H8	120.2	C20—C19—H19A	109.7
C8—C9—C10	120.78 (19)	O5—C19—H19A	109.7
C8—C9—H9	119.6	C20—C19—H19B	109.7
C10—C9—H9	119.6	O5—C19—H19B	109.7
C9—C10—C11	119.7 (2)	H19A—C19—H19B	108.2
C9—C10—H10	120.2	C19—C20—H20A	109.5
C11—C10—H10	120.2	C19—C20—H20B	109.5
O1—C11—C10	124.65 (18)	H20A—C20—H20B	109.5
O1—C11—C6	115.03 (17)	C19—C20—H20C	109.5
C10—C11—C6	120.32 (18)	H20A—C20—H20C	109.5
O1—C12—H12A	109.5	H20B—C20—H20C	109.5
O1—C12—H12B	109.5	C11—O1—C12	118.66 (17)
H12A—C12—H12B	109.5	C15—O3—C16	116.32 (19)
O1—C12—H12C	109.5	C18—O5—C19	117.7 (2)
H12A—C12—H12C	109.5	C2—S1—C3	91.58 (10)
			
C4—C1—C2—S1	0.5 (2)	S1—C3—C13—C14	−10.9 (3)
C13—C3—C4—C1	177.77 (18)	C3—C13—C14—C18	178.9 (2)
S1—C3—C4—C1	0.3 (2)	C3—C13—C14—C15	0.9 (3)
C13—C3—C4—C5	−1.4 (3)	C13—C14—C15—O2	103.5 (3)
S1—C3—C4—C5	−178.86 (14)	C18—C14—C15—O2	−74.5 (3)
C2—C1—C4—C3	−0.5 (3)	C13—C14—C15—O3	−74.7 (2)
C2—C1—C4—C5	178.72 (18)	C18—C14—C15—O3	107.2 (2)
C3—C4—C5—C6	110.5 (2)	C13—C14—C18—O4	−8.6 (3)
C1—C4—C5—C6	−68.6 (2)	C15—C14—C18—O4	169.6 (2)
C4—C5—C6—C7	96.2 (2)	C13—C14—C18—O5	171.06 (18)
C4—C5—C6—C11	−81.9 (2)	C15—C14—C18—O5	−10.8 (3)
C11—C6—C7—C8	0.4 (3)	C10—C11—O1—C12	−3.5 (3)
C5—C6—C7—C8	−177.7 (2)	C6—C11—O1—C12	176.92 (18)
C6—C7—C8—C9	−0.2 (4)	O2—C15—O3—C16	−4.3 (3)
C7—C8—C9—C10	0.3 (4)	C14—C15—O3—C16	173.90 (19)
C8—C9—C10—C11	−0.4 (4)	C17—C16—O3—C15	−167.5 (2)
C9—C10—C11—O1	−179.0 (2)	O4—C18—O5—C19	−2.1 (4)
C9—C10—C11—C6	0.5 (3)	C14—C18—O5—C19	178.30 (19)
C7—C6—C11—O1	179.11 (18)	C20—C19—O5—C18	95.8 (3)
C5—C6—C11—O1	−2.8 (3)	C1—C2—S1—C3	−0.25 (18)
C7—C6—C11—C10	−0.5 (3)	C4—C3—S1—C2	−0.03 (16)
C5—C6—C11—C10	177.62 (19)	C13—C3—S1—C2	−177.60 (17)
C4—C3—C13—C14	171.9 (2)		

### Hydrogen-bond geometry (Å, °)

**Table:**

*D*—H···*A*	*D*—H	H···*A*	*D*···*A*	*D*—H···*A*
C7—H7···O2^i^	0.93	2.55	3.429 (3)	159.

Symmetry codes: (i) −*x*+1/2, *y*−1/2, *z*.
